# A Review of the Treatment of the Principal Diseases at the German Hospital, Philadelphia

**Published:** 1875-09

**Authors:** Arthur Williams

**Affiliations:** Formerly Resident Physician


A Review of the Treatment of the Principal Diseases at the German Hospital, Philadelphia.—*By Arthur Williams, M.D., formerly Resident Physician.*—This institution, though of recent origin, bids fair to hold a prominent position among American hospitals. Many of its sister institutions can boast of greater wealth and capacity of accommodation, yet few can claim greater rapidity in general advances and improvement. Its various wards have been, from the first, well patronized, and embrace among their records the greater number of diseases common to this continent.

Patients suffering from the following diseases have been equally selected from each member of the medical staff, thus enabling us to give a review of the treatment adopted in each class of diseases.

PLEURITIS.

Most of the cases of simple acute pleurisy were treated by a blister to the side or sides affected, during the primary stage, combined with some anodyne, generally compound powder of ipecac. Where the acute symptoms had subsided, and the effused fluid was being very slowly absorbed, a series of blisters were commonly applied over the accumulation. Any debility that accompanied or followed the disease was counteracted by tonics, metallic and vegetable, with an ample and nutritious diet, aided by other hygienic influences. If to maintain a normal healthy condition of the bowels purgatives were required, they were used.

There were no cases of empyema admitted during our residence at the hospital, nor do the records furnish many. The course of treatment pursued was to sustain the patient by tonics, stimuli, and hygienic influences best adapted to individual cases. In no instance was an operation performed upon the chest.

BRONCHITIS—ACUTE AND CHRONIC.

In acute cases of a mild type, the patients were allowed the freedom of the ward, and usually placed upon some mild diaphoretic expectorant; for example,

℞.—Spirit Ætheris Nitrosi, . . . . . fl.ʒiss.

Liq. Potassæ Citratis, . . . . . fl.℥j.

Syrup Ipecacuanhæ, . . . . . fl.℥j.

Ft. sol.

Sig.—A teaspoonful every two hours.

During the second stage, should the cough still remain and become harassing, with profuse expectoration, a more stimulating expectorant was used, consisting of syrup of squill and senega with a little sulphate of morphia or hydrocyanic acid. If there was debility, the patient was given some mild tonic, with a small quantity of wine, or one of the malt liquors, at meals.

Chronic bronchitis was variously treated by inhalations and agents adapted to the general system; those of the latter class embracing chlorate of potassa, muriate of ammonia, iodide of potassium, and bromide of potassium and ammonium. In some of the cases one or more of the articles were used for a long time, in others variously interchanged, producing sometimes marked improvement, but again no perceptible benefit. The inhalations consisted of simple watery vapor, or this variously medicated. The following embrace those formulæ more commonly used:

℞.—Tinct. Conii,

Tinct. Hyoscyami, . . . . . aa. fl.ʒj.

Potassæ Chloratis, . . . . ʒj.

Aquæ, . . . . . . . fl.℥iv.

Ft. sol.

Sig.—Use as an inhalation, ter die.

℞.—Tinct. Opii Camphoratæ, . . . fl.ʒij.
Acidi Gallici, . . . . . . gr.xvj.
Aquæ, . . . . . . . . fl.℥iv.
Ft. sol.
Sig.—Use as an inhalation, ter die.

℞.—Liq. Potassæ arsenitis, . . . . gtt.xxxij.
Tr. Hyoscyami, . . . . . fl.ʒj.
Aquæ, . . . . . . . fl.℥iv.
Ft. sol.
Sig.—Use as an inhalation, ter die.

If the patient became weak and debilitated, tonics and a liberal diet were immediately ordered.

PNEUMONITIS.

All cases of asthenic type, when brought into the hospital, if in the first stage, and attended by severe pain, were given a full dose of opium, or some of its preparations. If a blister was deemed requisite, which was not often the case, it likewise was at once applied, extending over a great portion of the area of the diseased lung. In one case, that of a strong and vigorous youth, sixteen years old, with pneumonia of the lower lobe of the left lung, instead of the opiate and blister, or expectant plans, being followed, the ice treatment, as recommended by Niemeyer, was tried. A large bag of ice was applied over the diseased lung, and allowed to remain for three hours; this acted most kindly, removing all pain and dyspnœa at once. The ice was reapplied on the two following days, about the same hour, and for nearly the same length of time, after which the pulse resumed its normal character, and the pain and dyspnœa did not return. The case rapidly convalesced, and required no other treatment than a small quantity of wine and a little tincture of the acetate of iron, to remove some slight debility and increase the appetite.

In no case was blood drawn, either generally or locally. If the patient required depletion, purgatives were relied upon, in preference to blood-letting. For high febrile action, large doses of sulphate of quinia, and digitalis in some form, were used. The latter article often in the shape of the following recipe:

| | |
|---|---|
| ℞.—Liq. Ammoniæ Acetatis, . . | fl.℥j. |
| Spt. Ætheris Nitrosi, . . . | fl.℥ss. |
| Tinct. Digitalis, . . . . | fl.ʒj. |
| Aquæ, . . . . . . | fl.℥iss. |

Ft. sol.

Sig.—A teaspoonful every two hours.

Numerous cough mixtures were used to palliate the cough when it became painful and harassing. During convalescence, if the patient became debilitated, some mild ferruginous or vegetable tonic was used, combined with a liberal and nutritious diet.

Asthenic cases were put on a stimulating treatment from the first, embracing tonics, vegetable and mineral, with a nutritious, stimulating diet, and if requisite, alcoholic stimulants in some form. A common practice was to envelop the whole chest of the patient in a flaxseed poultice, and cover it with oiled silk. These patients were sometimes blistered, but never when the system was in a very low condition. If the patient became very much depressed, in addition to the alcoholic stimulants, carbonate of ammonia was used by the mouth, and in combination with oil of turpentine by enema; every endeavor being made to sustain the system until nature rallied sufficiently to carry on its vital functions.

PHTHISIS PULMONALIS.

Patients suffering from this disease, regardless of its special nature, were placed under treatment and influences calculated to increase the general tone and vigor of the system. Their diet was of the most nutritious character, liberal in amount, and if the patient was very much enfeebled, it was given at short intervals, combined with a small quantity of some alcoholic drink. The malt liquors were generally preferred, provided they did not produce digestive derangements. Should this be the case, then some of the lighter wines were resorted to, and even the stronger combined, or in alternation with whisky.

One of the medical staff placed all the phthisical patients on raw beef, minced and seasoned to suit the individual's taste. Each patient received four ounces of this for dinner and supper, additional to the general diet of vegetables and bread, and while eating was directed to drink two drachms of diluted alcohol, as prepared in the following formula:

| | |
|---|---|
| ℞.—Alcohol Dilut., . . . . . | fl.ʒij. |
| Syrup Aurantii Corticis, . . . . | fl.℥j. |
| Aquæ, . . . . . . . . . | fl.℥ij. |

Ft. sol.

Sig.—To be drank during one meal, while eating.

Cod-liver oil was used only in those cases where it was digested and assimilated. Iron, quinia, and the simple vegetable bitters were used in various combinations in nearly every case. Where there was an abnormally high temperature, with a rapid pulse, quinia, in large doses, either alone or combined with digitalis, was resorted to, and generally with the gratifying result of a return to the normal healthy condition. Whenever the cough became annoying, various cough mixtures were used to alleviate it; also inhalations of anodyne substances; for example, tincture of opium, simple and camphorated, or tincture of conium and hyoscyamus. To check the slight hæmoptysis incident to the course of this disease, reliance was chiefly placed upon inhalation of gallic acid; should this not prove sufficient, it was then administered by the mouth. Other astringents, such as ergot, tannic acid, and acetate of lead, were used in large doses for the same purpose.

### DIGESTIVE ORGANS.

#### GASTRO-ENTERITIS.

To restrain the vomiting, which was often persistent, the patient was placed at rest in the recumbent posture, and given small fragments of ice, and directed to swallow them undissolved. If this did not restrain it, other agents were resorted to, such as subnitrate of bismuth and tannic acid, in powder, camphor water, oxalate of cerium, and chalk mixture. Little or no food was allowed by the mouth, the patient being sustained by nutritious enemas. By this means the stomach was placed entirely at rest, and all sources of irritation removed. To prevent purging, from the enteric complication, the patient was first given a small dose of castor oil, to remove all feculent matter from the bowel, and then a dose of opium, combined, or not, with some astringent agent calculated to restrain their action. The following formulæ were very much used:

℞.—Confectionis Opii, . . . . . gr.xxxvj.

Misturæ Cretæ, . . . . . . fl.℥ij.

Tincture Kino, . . . . . . fl.ʒiij. M.

Ft. sol.

Sig.—A tablespoonful three times per day.

℞.—Aquæ Camphoris, . . . . . fl.℥ij.
Morphiæ Acetatis, . . . . . gr.ij.
Acidi Tannici, . . . . . . gr.x.
Ft. sol.
Sig.—A teaspoonful every two hours.

In several cases a blister was applied over the small intestines; in two instances it produced a marked decrease of all the symptoms, the pain subsiding to a great extent, and likewise the griping.

#### DYSENTERY—CHRONIC AND ACUTE.

In acute sporadic dysentery, the patient was put at rest in bed, and given some mild purgative, either castor oil or one of the salines. After the bowels had been evacuated, a full dose of some opiate preparation was given, to restrain the evacuations, relieve the tenesmus, and procure rest. If this did not suffice, the saline was repeated and another dose of opium given. If the patient desired it, small fragments of ice were allowed him, at liberty. Injections of cold water into the rectum, though at first painful, in a short time afforded great relief, decreasing the number of evacuations, and to a large degree removing the tenesmus. If after checking the dysentery there remained any debility, it was removed by tonics, healthy exercise, and a good diet. Those patients suffering from a chronic form of dysentery were generally much debilitated when they came under observation, and consequently required a sustaining treatment. They received a liquid but very nutritious diet, and tonics, if they were required. For the local disease of the bowel, various agents and methods were resorted to. A pill of opium and nitrate of silver, containing a quarter of a grain of each, was given in most cases, for two weeks or more, but we cannot recall a single instance where its use produced any decided benefit. A powder of subnitrate of bismuth and pepsin, containing ten grains of the former to five of the latter, was often used, giving three or four per day, according to the requirements of the case. Suppositories of opium were used. They checked the evacuations for the time, and often for some time after their administration. More attention was given to improvement of the patient's general condition than to local measures.

DIARRHŒA.

Simple diarrhœa was treated by astringents of various characters. The most simple and frequently used was chalk mixture, or this in combination with confection of opium and tincture of kino or krameria. Subnitrate of bismuth and tannic acid were also used, the latter combined with simple or camphorated tincture of opium. After the diarrhœa was stopped, the diet was regulated for some time, consisting mostly of bland and easily digested articles. If any debility remained, tonics were given until it was removed.

PERITONITIS.

Patients with simple acute peritonitis, as soon as admitted, were put under the influence of opium, and kept so until the acute symptoms subsided. Warm fomentations were applied to the abdomen, such as warm turpentine, cloths wrung from hot water, and very light poultices. A liquid diet of milk punch, beef tea, soup, and such articles only, was allowed. During convalescence, if tonics or laxatives required, they were used.

PERIHEPATITIS.

These cases were put to bed and given eight or ten grains of calomel at night, following it in the morning by a full dose of sulphate of magnesia, or fluid extract of senna and rhubarb, in equal proportions. If necessary, the doses of calomel and purgative were repeated. A blister was generally applied over the seat of inflammation; if this did not remove the pain, the patient was given opium or chloral at night, to secure rest. If any other symptoms arose during the attack, they received proper attention.

No cases of hepatitis are mentioned in the records of the hospital, nor were any present during our residence there. Hepatic congestion, however, occurred quite frequently. This was treated by a mild course of purgatives, into which some mercurial preparation entered. A mild, unstimulating diet was directed, and alcohol in every form positively prohibited. If during convalescence the appetite was much impaired, some one of the simple vegetable bitters was given.

NERVOUS SYSTEM.

CEREBRO-SPINAL MENINGITIS.

There are no cases of simple, acute and tubercular meningitis mentioned, but several of cerebro-spinal meningitis. In the latter, ice was applied to both the head and the spine, accompanied with full doses of opium, until the acute symptoms began to subside, when ice was discontinued, and the opium decreased in amount. In only one case was quinia used during the first stage. For the debility which nearly always attended and followed the disease, iron, quinia and some of the vegetable bitters, variously combined, were used.

DELIRIUM TREMENS.

In those cases of the disease presenting marked hallucinations, and even delirium, the patient was given large doses of the bromides, twenty or thirty grains, three times per day. In other cases opiates, subnitrate of bismuth and small quantities of alcohol, in some palatable form, were used, instead of the bromides.

An infusion of hops was used in large doses and with some advantage. Under its use the patient became calm, less restless, and inclined to sleep. As a stimulant to the alimentary mucous membrane, pills of capsicum, of five or eight grains, and aromatic sulphuric acid, in doses of fifteen drops, three times per day, were used.

Where the attack was dependent upon a severe and recent debauch, the treatment varied somewhat from the above. As the chief symptom to be counteracted here was cerebral congestion, purgatives and the bromides were preferable to opium. The following is a favorite purgative mixture of one of the staff:

| | |
|---|---|
| ℞.—Sodæ et Potassæ Tartratis, . . . | ʒiij. |
| Spiriti Ætheris Nitrosi, . . . . | flʒiss. |
| Extracti Senæ Fluidi, | |
| Extracti Rhei Fluidi, . . . . | aa fl.ʒij. |
| Tincture Hyoscyami, . . . . | fl.ʒij. |
| Aquæ menthæ piperitæ, . . . | fl.℥iij. |

Ft. sol.

Sig.—A tablespoonful every two hours.

The food was chiefly liquid, consisting of soups, milk, beef essence, etc. During convalescence the patient's general condition was improved by tonics, and as much good food as he could thoroughly digest.

### APOPLEXY.

An expectant plan of treatment was followed almost exclusively in this disease. In no instance was blood-letting employed. The patients were usually brought into the hospital after the primary symptoms were over with; hence a resort to this measure was not called for. If the action of the bowels was impaired, purgatives were given at proper intervals to remove the fecal matter. Small quantities of iodide of potassium, compound tincture of gentain, or compound tincture of cinchona, were used as placebos, and the symptoms treated separately as they arose.

### EPILEPSY.

Epileptic patients were mostly given bromides, in large doses and long continued. Some were placed upon the following powder, for a long time without intermission:

℞.—Sodæ Hyposulphis, . . . . . . ℈

Pepsin, . . . . . . . . ʒss.

Morphiæ Sulphatis, . . . . . . gr. $\frac{1}{4}$.

Ft. pulv., viij.

Sig.—One after each meal.

Others, again, were put on a powder of oxide of zinc and powdered belladonna, two grains of the former to one-quarter of a grain of the latter; receiving one every three hours. The patient's general condition received proper attention; if debilitated, tonics were given—if constipated, laxatives.

HYSTERIA.

Hysterical patients were given one of the bromides in full medicinal doses; if this did not exercise a controlling influence over the paroxysms, then some of the anti-spasmodics, valerian, asafœtida, or compound spirits of ether, were tried. If the patient was anæmic, tonics and a liberal diet were given. All menstrual derangements were given close attention. If the patient suffered from suppressio mensium, every effort was made to establish that function by tonics, stimulating purgatives, aloetic or otherwise, hot hip-baths at every revolution of the menstrual period, and cold to the spine. The following mixtures, combining anti-spasmodic and purgative properties, well adapted to constipation when occurring in this disease, were used in many of the cases:

℞.—Infusi Valerianæ, . . . . . . fl.℥ij.

Tincture Hyoscyami, . . . . . . fl.ʒij.

Extracti Senæ Fluidi, . . . . . fl.ʒij.

Tincture Rhei, . . . . . . . fl.℥ss.

Ft. sol.

Sig.—A tablespoonful every three hours.

℞.—Ext. Sennæ Fluidi, . . . . . fl.℥ss.

Spt. Æth. Chlorici, . . . . . . fl.℥ij.

Liq. Ammoniæ Acetatis, . . . . . fl.℥j.

Aquæ Camphoræ, . . . . . . fl.℥ij.

Syrupi Auranti Corticis, . . . . . fl.℥ss.

Ft. sol.

Sig.—A tablespoonful every three hours.

FEVERS.—TYPHOID FEVER.

Cases of typhoid fever, as soon as admitted, were put in bed and at complete rest. They were not permitted to get up for any purpose whatever. Their food was liquid in character, and given in small quantities and at short intervals. If the patient's condition required it, alcohol was used moderately from the first, increasing the amount with the advance of the case, as there was a demand for it. Sulphate of quinia was used in most of the cases, the amount often reaching fifteen and twenty grains per day. The following formulæ were often used for its administration:

| | |
|---|---|
| ℞.—Quiniæ Sulphatis, . . . . | gr.xvj. |
| Acidi Muriatici, . . . . . . | gtt.xvj. |
| Syrupi Aurantii Corticis, . . . | fl.℥iij. |
| Tincturæ Gelsaminii, . . . . | fl.ʒij. |

Ft. sol.

Sig.—A tablespoonful every three hours.

| | |
|---|---|
| ℞.—Pulvis Cinchonæ, . . . . | ℥j. |
| Pulvis Serpentariæ, . . . . . | ʒij. |
| Sodæ Bicarbonatis, . . . . | fl.℥xij. |

Mix and filter.

Sig.—fl.℥j, ter die.

After the disease was somewhat advanced, and ulcers were forming or formed in the bowel, oil of turpentine, in doses of ten or fifteen drops, in emulsion, was given three or four times per day, and continued into convalescence.

MALARIAL FEVER.

In mild cases the treatment consisted in administering sulphate of quinia, in four or five grain doses, until fifteen or twenty grains was reached, to stop the paroxysm. If this did not prove successful, then the dose was repeated once or twice more, until the paroxysm was checked. Along with, or before administering the quinia, five or more grains of blue mass were given at night, and followed in the morning by some mild aperient.

Where the case was of greater gravity, the treatment varied somewhat from that of the milder form. The sulphate of quinia was used in the same quantity, and repeated as often as required. Likewise, a mild mercurial purge preceded or followed the quinia; but if there was vomiting, ice was given in small fragments, and swallowed undissolved, or the ice could be combined with or substituted by subnitrate of bismuth. If there was much headache, the patient had ice, or a cloth wrung out of ice water, applied to the head. After the paroxysm was stopped, the patient was put on a mixture of iron and quinia, and kept under its influence for several weeks, to prevent the relapse of the disease.

ERYSIPELAS.

The treatment of this disease differed considerably with the individual members of the staff. Internally, some gave diaphoretics, such as neutral mixture, solution of acetate of ammonia and spirit of nitrous ether; others, simply oil of turpentine in emulsion, if there was suppression of the secretions. Iron was used in nearly all the cases, chiefly as a tincture of the chloride. A non-official preparation, known as an elixir of strychnia, quina and iron, was used to some extent. If the patient was constipated or had diarrhœa, either an astringent or purgative was given as required.

As local dressings, dry cotton, flexible collodion, hyposulphite of soda in glycerine, and tincture of iodine were used.

RHEUMATISM.

Several methods of treatment were employed in this disease. As a local dressing, dry cotton was often used, or this dipped in a solution or sprinkled with the powdered nitrate of potassium. Likewise, belladonna plaster and ointment were used for the same purpose, completely enveloping the joint with them. Some preference was given, by one of the staff, to the following liniment, as a local application to the inflamed joint.

℞.—Linamenti Chloroformis.
Linamenti Saponis Camphorati, . . . aa fl.℥j.
Tincturæ Aconiti Radicis, . . . . . fl.ʒj.
Ft. sol.
Sig.—Use as a liniment.

The constitutional treatment addressed directly to the disease consisted chiefly of the alkaline carbonates, and bicarbonates in various combinations, either with or without colchicum, as embraced in the following formula.

℞.—Potassii bicarbonatis,
Sodii Bicarbonatis, . . . . . aa ʒj.
Vini Colchici Seminis, . . . . . gtt.xl.
Aquæ, . . . . . . . . . Oss.
Ft. sol.
Sig.—A wineglassful every two hours.

The bromides were used, but not very extensively. As aperients, Scudamore's mixture and the following recipe were constantly used:

℞.—Potassii bromidi, . . . . . . ℨiij.

Vini colchici Seminis, . . . . . . fl.ℨj.

Extracti Sennæ Fluidi, . . . . . fl.℥iv.

Aquæ, . . . . . . . . . fl.℥iij.

Ft. sol.

Sig.—A tablespoonful every three hours.

Many of the patients simply had the affected portion wrapped in dry cotton, and given a mild aperient. Others, again, where much debilitated, were given, additional to this, some tonic ferruginous mixture, and its use continued until convalescence was well established.

BURNS AND SCALDS.

In the greater portion of these cases, equal parts of linseed oil and lime-water, mixed, was used as local dressing. In some few glycerine or mucilage of slippery elm was used. Recently a mixture of glycerine, hyposulphite of soda and chloroform has been used several times with rather pleasing results. The constitutional treatment consisted in sustaining the patient's system against collapse, by alcoholic stimulants, alleviating pain by anodynes, and, if any debility followed in the latter stages, tonics and a nutritious diet.

ABSCESS—ACUTE AND CHRONIC.

Acute abscesses were poulticed until brought to a head, the pus evacuated, and the remaining wound treated with a simple water dressing.

In chronic abscess, the patient was given tonics, a small quantity of alcohol, with nutritious food, and placed under all influences calculated to invigorate the system. After a considerable quantity of pus had accumulated, it was evacuated, either by the aspirator or a free incision. If an incision was made, a drainage tube was put in the sac, and the pus permitted to drain away as fast as formed. The cavity of the abscess was washed out several times per day with either tepid water, a solution of permanganate of potash, or a dilute solution of carbolic acid.

SYPHILIS.

In primary syphilis, if chancres were present, but did not present an unhealthy appearance, they were simply treated by a lead-water and laudanum lotion. Should they require slight stimulation, a solution of sulphate of zinc or copper, two or three grains to the ounce, was used. If it was sloughing and disinclined to put on a healthy appearance, or if indurated, the part was thoroughly cauterized by nitric acid, caustic potash, or some other suitable caustic. After the eschar separated, the ulcer remaining was dressed as the preceding, or with black or yellow wash.

The constitutional treatment depended very much upon the condition of the patient. If broken down and emaciated, he was put on a course of tonics, embracing iron in some form, more commonly the tincture of the chloride or the syrup of the iodide. Along with this tonic course, iodide of potassium was used in most of the cases, in doses of fifteen grains three times per day. If the patient's system had not been broken down, or if much lowered had been brought up to a good standard, he was at once put on mercury again, or murcury and iodide of potassium combined. The mercurials most commonly used were the corrosive chloride and green iodide; the former in doses of one-sixteenth of grain, combined with fifteen grains of iodide of potassium, three times per day; and of the latter three grains three times per day.

GONORRHŒA.

For the acute stage, rest and injections of lukewarm water, and the following recipe, constituted the treatment:

| | |
|---|---|
| ℞.—Potassi Bromidi, . . . . . | ℨiss. |
| Glycerin, . . . . . . . | fl.ℨijss. |
| Aquæ, . . . . . . . . | fl.℥iv. |

Sig.—Use as an injection twice daily.

After the acute symptoms had subsided somewhat, other injections were used, such as dilute solutions of sulphate of copper and zinc, containing two grains to the ounce. Some of the staff at this stage used an emulsion of balsam of copaiba internally. If there was danger of epididymitis or persistent inflammation, the patient's diet was restricted for some time, and saline purges given. Often the acute symptoms merged into a chronic form, with a persistent gleety discharge. When this was the case, the strength of the zinc and copper solutions was increased, or the injection changed for one consisting of acetate of lead and zinc, each six grains to eight ounces of water.

If epididymitis resulted, the patient was put at rest on his back, the testicles supported on a cushion, and cooling lotions applied, if there were acute inflammatory symptoms. Should the epididymis become chronically indurated and indisposed to soften, then mercury was applied locally, either in the form of the simple ointment, or of that combined with belladonna ointment, in the proportion of eight of the former to two of the latter. In place of the mercurial preparation, an ointment containing iodine was sometimes used. The following formula was one of the most common:

℞.—Unguenti Iodini, . . . . ℥ij.

Extracti Belladonnæ, . . . . gr.xx.

Adipis, . . . . . . ℥ij M.

Ft. unguentum.

Sig.—Apply externally twice per day.

BUBO.

If the bubo had not attained a large size, an effort was made to dissipate it. The patient was put at rest, and some applications made to the gland. The more common of these were tincture of iodine, ointment of iodide of lead, ointment of iodine simple and compound, and ointment of mercury and belladonna combined. Pressure, by means of a weight or compress, was often resorted to. If the efforts at dissipation were not successful, poultices were at once applied, and, after pus had formed, the gland laid freely open and evacuated of its contents. The remaining ulcer was made to heal by granulation from the bottom. If it became indolent and indisposed to heal, it was touched from time to time with crystal of sulphate of copper or nitrate of silver. If a milder application than this only was required, then a weak solution of sulphate of zinc and copper was used.

